# Interplay between lipid lateral diffusion, dye concentration and membrane permeability unveiled by a combined spectroscopic and computational study of a model lipid bilayer

**DOI:** 10.1038/s41598-018-37814-x

**Published:** 2019-02-06

**Authors:** Muhammad Jan Akhunzada, Francesca D’Autilia, Balasubramanian Chandramouli, Nicholus Bhattacharjee, Andrea Catte, Roberto Di Rienzo, Francesco Cardarelli, Giuseppe Brancato

**Affiliations:** 1 Scuola Normale Superiore, piazza dei Cavalieri 7, I-56126 Pisa, Italy; 2 Istituto Nazionale di Fisica Nucleare, Largo Pontecorvo 3, I-56100 Pisa, Italy; 3 Center for Nanotechnology Innovation@NEST (CNI@NEST), Pisa, Italy; 4 Dipartimento di Ingegneria dell’Informazione, Università di Pisa, Via Girolamo Caruso 16, I-56122 Pisa, Italy; 5 NEST, Scuola Normale Superiore and Istituto Nanoscienze-CNR, Piazza San Silvestro 12, 56127 Pisa, Italy; 6 Present Address: Compunet, Istituto Italiano di Tecnologia (IIT), Via Morego 30, I-16163 Genova, Italy

## Abstract

Lipid lateral diffusion in membrane bilayers is a fundamental process exploited by cells to enable complex protein structural and dynamic reorganizations. For its importance, lipid mobility in both cellular and model bilayers has been extensively investigated in recent years, especially through the application of time-resolved, fluorescence-based, optical microscopy techniques. However, one caveat of fluorescence techniques is the need to use dye-labeled variants of the lipid of interest, thus potentially perturbing the structural and dynamic properties of the native species. Generally, the effect of the dye/tracer molecule is implicitly assumed to be negligible. Nevertheless, in view of the widespread use of optically modified lipids for studying lipid bilayer dynamics, it is highly desirable to well assess this point. Here, fluorescence correlation spectroscopy (FCS) and molecular dynamics (MD) simulations have been combined together to uncover subtle structural and dynamic effects in DOPC planar membranes enriched with a standard Rhodamine-labeled lipid. Our findings support a non-neutral role of the dye-labeled lipids in diffusion experiments, quantitatively estimating a decrease in lipid mobility of up to 20% with respect to the unlabeled species. Moreover, results highlight the existing interplay between dye concentration, lipid lateral diffusion and membrane permeability, thus suggesting possible implications for future optical microscopy studies of biophysical processes occurring at the membrane level.

## Introduction

Plasma membrane not only provides the necessary compartmentalization to protect the cell, but is directly involved in a variety of vital cellular processes such as signaling, transport of biomaterial, cell adhesion, etc…^1^. To comply with such a variety of roles, the plasma membrane requires a high degree of structural plasticity. Dynamic lateral assembly of lipid/protein complexes combined with a regulated compositional patterning of lipids on both membrane leaflets provide cells with the opportunity to decorate this interface with specific molecules in an organized but dynamic manner. For such reasons, in recent years a great effort has been directed towards the understanding of lipid diffusion and self-organization at a molecular level. Among others, fluorescence-based optical microscopy methods have gradually emerged as versatile, quantitative tools to investigate the complex spatiotemporal organization of lipid membranes in both model bilayers and living cells: both localization-based (e.g., single-particle tracking, SPT) and statistical methods (e.g., fluorescence correlation spectroscopy, FCS), eventually combined with super-resolution approaches, greatly contributed to build the current knowledge on this topic (see refs^2–6^).

Irrespective of the particular technique chosen, however, a common requisite for optical microscopy measurements is labelling the molecule of interest with a fluorescent tracer, or probe. In studies on lipids, in particular, optical probes are typically chosen as lipid analogs^7^, such as the dialkylcarbocyanines, or dye-labeled lipids, such as Bodipy, Rhodamine or Atto^8^. In the typical configuration of a comparative study, the measured dynamics of the labeled lipid will depend, and provide information, on several crucial aspects, such as the water content^9^, the aqueous phase composition (ionic strength and other soluble species, like sugars), the specific lipid composition (i.e., mixture of lipids and/or sterol components) and other physical/mechanical conditions, such as temperature, pressure, membrane tension, etc. Moreover, these investigations will also provide information on the lipid phase, including the possible co-existence of liquid-disordered (*L*_*d*_), liquid-ordered (*L*_*o*_) or raft-like domains.

Still, in all these optical microscopy studies, the implicit assumption is made of ignoring the perturbing effect of the probe, based on the consideration that any spurious effect, if present, is irrelevant with respect to the physico-chemical properties under study. While this is perhaps a reasonable assumption to make, especially in comparative studies involving systems of variable chemical compositions, it is worth noting that optical probes have often shown a non-neutral role when tagged to biomolecules, as it was recently reported by some of us in the context of cell-penetrating peptides and their interaction with biomembranes^10,11^. Along the same line, previous studies in which different optical probes have been tested in the same lipid bilayer systems, have reported either different^12,13^ or comparable^14^ lipid diffusion properties, making the interpretation of the results not always straightforward. Besides, diffusion measurements based on dye-labeled lipids have been shown to depend on lipid bilayer compositions, but also on the dye concentration^15^.

**Figure 1:**
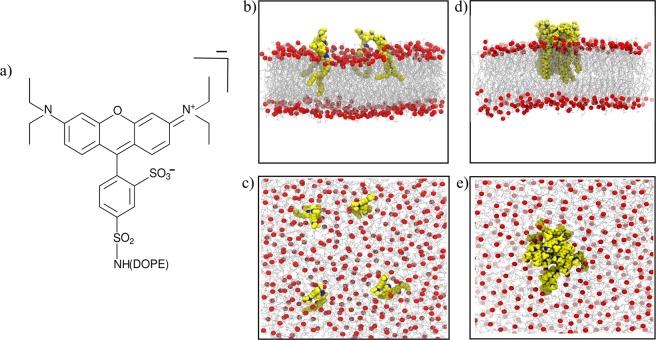
(**a**) Scheme of the RHB lipid. (**b**) Side and (**c**) top view of a MD simulation snapshot of the RHB-DOPC lipid bilayer. (**d**) Side and (**e**) top view of a MD simulation snapshot of the RHB-DOPC bilayer displaying RHB self-aggregation. RHB lipids are shown in yellow, with nitrogen in blue, while DOPC lipids are in gray with corresponding phosphate atoms in red; water is omitted. In (**b**–**e**), only RHB lipids in one leaflet are displayed for the sake of clarity.

Based on these considerations, it appears desirable to better assess the role of a tracer molecule when exploited for studying lipid diffusion in planar membranes. In this context, molecular dynamics (MD) simulations represent a valuable tool to gather structural and dynamic features of complex biosystems not easily accessible from experiments^16–18^. However, care has to be taken in order to fruitfully exploit MD techniques, since previous studies have highlighted unwanted artifacts due to the limited timescale and the typical finite size of the atomistic models. For example, local lipid diffusion was shown to be affected by the system size in small bilayer models^19–21^ and displayed anomalous (non-Brownian) behavior when evaluated at short timescales (i.e., 1–10 ns)^22^. Thus, here we propose a combination of FCS experiments and extended atomistic MD simulations to shed light on some important features concerning lipid dynamics and lipid structural properties within a simple 1,2-dioleoyl-sn-glycero-3-phosphocholine (DOPC) bilayer. Through such an integrated approach, our study attempts at better assessing the possible direct and indirect effects of a typical dye-labeled lipid, here Rhodamine B linked to 1,2-dioleoyl-sn-3-phosphatidylehanolamine (hereafter referred to as RHB, see scheme of the molecule in Fig. 1a), on the estimation of the lipid diffusion coefficient in a planar membrane. To this end, giant unilamellar vesicles (GUVs) of DOPC with increasing concentration of RHB (from 10^−4^% to 10%) have been produced, since they are considered a more realistic membrane model than supported lipid bilayers (SLBs), and an *in silico* molecular model of up to 400 lipids has been considered in our complementary computational investigation, see Fig. 1b,c. In particular, we evaluated the hydrodynamic effect of the Rhodamine B fluorophore and investigated the effects of RHB concentration on the structural and dynamic properties of the lipid bilayer. Results have shown a non-negligible role of the tracer molecule, which induced a decrease of about 20% in the observed lipid lateral diffusion with respect to the unlabeled species. Moreover, we have characterized the effect of the dye-driven RHB self-aggregation with concentration and the relevant impact that the formed dye-labeled lipid clusters (Fig. 1d,e) have on both membrane structure and permeability.

## Results

### Structural properties of the RHB-DOPC bilayer

Structural properties of the DOPC membrane upon insertion of a dye-labeled lipid were investigated using atomistic MD simulations. A membrane with concentration of 2% RHB (corresponding to 8 lipids over a total of 400 lipids, 4 in each leaflet) was generated as described in the Methods. Note that RHB lipids were introduced in the DOPC bilayer in such a way to minimize self-interactions. Accordingly, we have observed no contacts occurring during the four replica simulations in the considered time interval (see Supplementary Fig. S1). First, we evaluated the membrane thickness and the average area per lipid as compared to the pristine DOPC membrane. Bilayer thickness was defined as the average distance between phosphate headgroups of both leaflets. Average thickness of the pure DOPC system was found to be 38.6 Å (Table 1), in well agreement with previous experiments (36.7–38.5 Å^22–24^), and displaying no significant difference with respect to the Rhodamine B-labeled bilayer: all RHB-DOPC systems have shown a membrane thickness of about 38 Å. Average area per lipid (ApL) is the most common determining feature in the packing of the lipid bilayer and structure. Pure DOPC system provided ApL of 68.2 Å^2^, close to recent experimental data and well within previously reported measurements at similar temperature (67.4–72.5 Å^2^ ^23–25^). On the other hand, RHB-DOPC systems have shown a slight increase in ApL (about 69 Å^2^). Area per lipid and membrane thickness for all systems are reported in Table 1.

**Table 1:** Structural and dynamic properties of the DOPC and RHB-DOPC bilayer systems as issuing from corresponding MD simulations.

System	ApL (Å^2^)	Thickness (Å)	D^DOPC^ (μm^2^ s^−1^)	D^RHB^ (μm^2^ s^−1^)
DOPC	68.2 ± 0.8	38.6 ± 0.4	8.4 ± 0.4	—
RHB-DOPC (Average over 4 replica)	69.2 ± 0.8	38.2 ± 0.4	7.8 ± 0.4	6.7 ± 0.7
RHB-DOPC (NaCl 0.5 M)	67.7 ± 0.8	38.5 ± 0.4	7.7 ± 0.7	6.0 ± 2.1
RHB^Agg^-DOPC (RHB self-aggregation)	68.7 ± 0.8	37.9 ± 0.4	8.7 ± 1.0	4.0 ± 1.2

Errors in ApL and thickness correspond to one standard deviation. Diffusion coefficients and corresponding uncertainties were evaluated according to the procedure described in Sec. 4.7.

**Figure 2:**
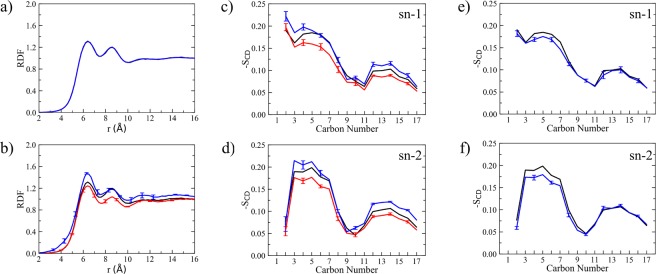
(**a**) P-P radial distribution functions (RDFs) obtained considering only DOPC-DOPC pairs as issuing from MD simulations of DOPC (black), RHB-DOPC (red) and RHB^Agg^-DOPC (blue, RHB self-aggregation) lipid bilayers. Note that the three lines appear as superimposed on one another. (**b**) P-P RDFs obtained from DOPC (black, DOPC-DOPC pairs), RHB-DOPC (red, RHB-DOPC pairs) and RHB^Agg^-DOPC (blue, RHB^Agg^-DOPC pairs) lipid bilayer simulations. (**c**,**d**) Deuterium order parameters (*S*_CD_) evaluated for lipid acyl chains (i.e., sn-1 and sn-2) as issuing from MD simulations of DOPC (black, DOPC lipids), RHB-DOPC (red, RHB lipids) and RHB^Agg^-DOPC (blue, RHB^Agg^ lipids) lipid bilayers. (**e**,**f**) Deuterium order parameters (*S*_CD_) from MD simulations of DOPC (black, DOPC lipids) and RHB^Agg^-DOPC (blue, only DOPC lipids within 5 Å from any RHB^Agg^ lipid) lipid bilayers. In all diagrams, error bars correspond to one standard error. Error bars of pure DOPC system (black lines) are negligible and then omitted for clarity in all plots.

Moreover, radial distribution functions (RDFs) of both native DOPC and Rhodamine B-labeled lipids were evaluated considering the common phosphate group (i.e., P atom). Results are reported in Fig. 2a,b. RDFs show two main peaks and a less pronounced third peak corresponding to successive lipid shells, where the first shell extends from 6.0 to 7.5 Å and the second one from 8.2 to 10 Å. Positions of the first two RDF peaks, which determine the probability of finding phosphate groups in the neighboring shells, are consistent throughout all lipid membrane simulations, although peak height is somewhat different in the two simulations as a result of local lipid rearrangements upon RHB insertion: in terms of P-P RDF, the lipid bilayer appears less structured around RHB lipids. In turn, this perturbation may affect the local DOPC structure in the second shell, but this is expected to be an even smaller effect. Note, however, that RHB-DOPC results are affected by larger noise with respect to DOPC ones, owing to poorer statistics (i.e., RHB lipids are two orders of magnitude less than DOPC lipids).

Furthermore, we calculated the deuterium order parameters, *S*_CD_, for both hydrocarbon chains (i.e., sn-1 and sn-2 chains) of the lipid tails in DOPC and RHB. Note that the chemical structure of the acyl chains is exactly the same in both lipids, with a double bond between carbon 9 and 10. The *S*_CD_ values can vary between 0 to 0.5, where smaller values indicate more flexible/disordered regions while higher values correspond to relatively more rigid/ordered regions^22,26^. The computed *S*_CD_, as depicted in Fig. 2c,d, show that the degree of flexibility of the lipid tails does increase when going towards the terminal parts, since the mobility is rather unrestricted in the middle of the bilayer, with the exception of the typical sharp dip caused by the presence of the double bond (i.e., C=C at 9–10) and the unusual small value of C2 in the second tail (i.e., sn-2). Overall, *S*_CD_ of DOPC matches well reported order parameters from previous computational^27^ and experimental^28^ studies. Concerning RHB, *S*_CD_ is observed to be rather similar to the unlabeled counterpart, as expected giving the identical chemical nature of the tail, although *S*_CD_ values do appear as consistently lower than DOPC (Fig. 2c,d). Hence, the present result suggests that RHB lipids are somewhat less ordered in comparison to DOPC. Nevertheless, the above structural analyses (i.e., thickness, ApL, RDF and *S*_CD_) have shown, overall, no large effects caused by the introduction of RHB in the DOPC bilayer.

### Lipid lateral dynamics: DOPC vs RHB-DOPC

**Figure 3:**
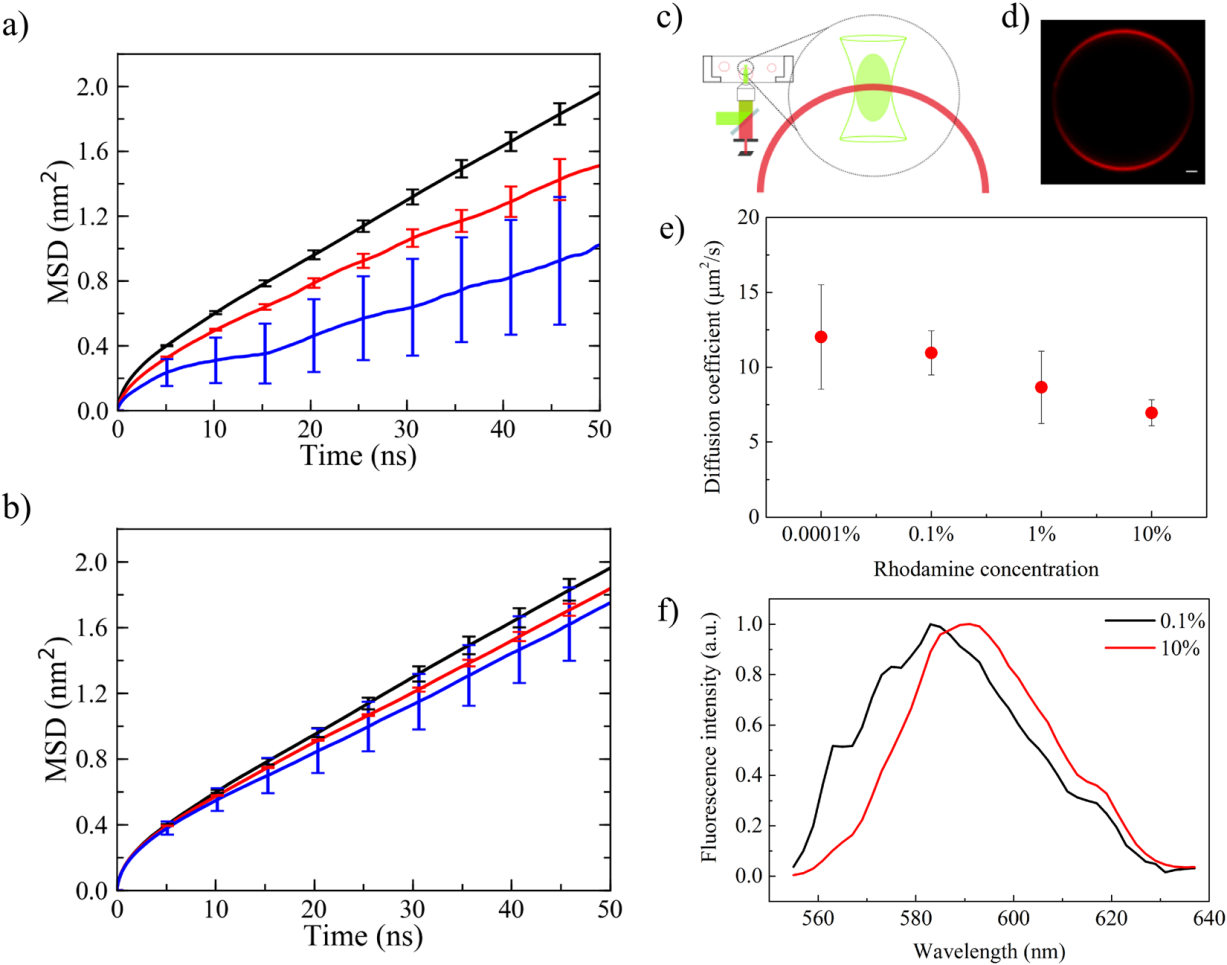
(**a**) Mean square displacement (MSD) evaluated as a function of time from MD simulations of DOPC (black, DOPC lipids), RHB-DOPC (red, RHB lipids) and RHB^Agg^-DOPC (blue, RHB^Agg^ lipids) lipid bilayers. Error bars correspond to standard errors. Differences in error bars reflects mainly the different number of systems (i.e., 400 DOPC lipids, 8 free RHB lipids, 1 RHB aggregate). (**b**) Mean square displacement (MSD) evaluated as a function of time from MD simulations of DOPC (black, DOPC lipids), RHB-DOPC (red, DOPC lipids) and RHB^Agg^-DOPC (blue, DOPC lipids) lipid bilayers. Error bars correspond to standard errors. (**c**) Schematic representation of a FCS measurement. (**d**) Confocal microscopy image of a GUV DOPC − 0.1% RHB, scale bar 1 micron. (**e**) Effect of fluorophore concentration on the diffusion of RHB lipids in GUVs. (**f**) Emission spectra of Rhodamine B at different concentrations (0.1% and 10%) in GUVs, the aggregation of Rhodamine B induces an emission spectra red shift of 8 nm, from 583 to 591 nm.

Lipid lateral dynamics for DOPC and RHB-DOPC membranes was analyzed in terms of mean square displacements (MSD) and diffusion coefficient as issuing from the corresponding MD simulations (see Methods for details). To increase the statistics, reported results represent the average over all four RHB simulations. MSD results are depicted in Fig. 3a,b. In the initial 10 ns, an anomalous non-linear increase of the MSD versus time was observed: this is due to a “cage effect” in the short timescale motions of the lipids, as previously reported^29^. Beyond 10 ns, the lipid dynamics becomes fully Brownian and it corresponds to the dynamical regime usually detected in fluorescence experiments at longer timescale (i.e., >microsecond). In each system, diffusion coefficient was then calculated by fitting the MSD as described in the Methods (Sec. 4.7), after skipping the first 10 ns of each trajectory. The obtained diffusion coefficients are reported in Table 1. The evaluated diffusion coefficient of DOPC, D = 8.4 ± 0.4 μm^2^ s^−1^, is in line with previous experimental and computational studies reporting a diffusion constant in the range 5–14 μm^2^ s^−1^, depending on temperature, relative water content and the nature of the planar membrane (e.g., GUVs vs SLBs)^9^. Besides, DOPC mobility was analyzed as issuing from the RHB-DOPC simulations and a very similar diffusion coefficient was obtained (7.8 ± 0.4 μm^2^ s^−1^). On the other hand, RHB displayed some noticeable slower dynamics (6.7 ± 0.7 μm^2^ s^−1^) with respect to the unlabeled lipid, thus reflecting, in our view, the larger hydrodynamic drag due to the bulky Rhodamine B headgroup. Moreover, we tested the effects of ion concentration on the lateral dynamics of the dye-labeled lipid considering the addition of 0.5 M NaCl. Results have shown a slight difference with salt concentration, within the estimated noise error, providing a diffusion coefficient of 6.0 ± 2.1 μm^2^ s^−1^ and a decrease in ApL (67.7 Å^2^). Both effects have been noticed in previous FCS and MD simulations studies^30,31^: upon NaCl salt addition, sodium ions penetrate into the headgroup region of the membrane, neutralize the negatively charged headgroups of the lipids and, by decreasing their mutual repulsion, increase their condensation, thus resulting in lower area per lipids. Note, however, that no sodium ion overbinding versus lipid phosphate groups was observed when comparing DOPC and RHB (see P-Na RDFs depicted in Supplementary Fig. S2; average number of sodium ions around each RHB or DOPC phosphate group within a radius of 0.6 nm was found to be 0.28, a result in well agreement with previous findings^31^).

### FCS measurements of lipid lateral diffusion: effects of RHB concentration

The lateral diffusion of RHB lipids at different concentrations was investigated in a parallel experimental setup. GUVs of RHB-DOPC in a ratio ranging from 10^−4^% to 10% have been prepared (see Methods for details). Immobilization of GUVs in agarose gel allowed performing FCS measurements on vesicle membranes (Fig. 3c,d). FCS was used to measure the diffusion of the dye-labeled lipid, RHB, across the membrane bilayer. Fluorophore concentration was high in vesicles and for this reason photobleach of Rhodamine B has been necessary. The measured diffusion coefficient of RHB lipid has shown a stark decrease with concentration as depicted in Fig. 3e: at very low concentration (10^−4^%), RHB diffusion is about 12 ± 3.5 μm^2^ s^−1^, and it becomes as low as 6.9 ± 0.9 μm^2^ s^−1^ at high concentration (10%) (Table 2).

**Table 2:** FCS measurements of RHB and LAURDAN, diffusion coefficients (D) and G_0._

Dye		DOPC	0.0001%	0.1%	1%	10%
RHB	D (μm^2^/s)	N.A.	12 ± 3.5	10.9 ± 1.5	8.7 ± 2.4	6.9 ± 0.9
	G_0_	N.A.	0.12 ± 0.06	N.A.*	N.A.*	N.A.*
LAURDAN	D (μm^2^/s)	12.2 ± 3.6	N.D.	10.1 ± 2.2	17.3 ± 5.1	24.8 ± 3.5
	G_0_	3.3 10^−3^ ± 1.9 10^−3^	N.D.	0.94 10^−3^ ± 0.5 10^−3^	3.7 10^−3^ ± 0.9 10^−3^	2.3 10^−3^ ± 0.5 10^−3^

^*^Data not available due to bleaching of Rhodamine B

A t-test analysis on the measured D values is reported in Supplementary Table S1 (RHB) and S2 (LAURDAN).

### High RHB concentration: aggregation and membrane permeability change

One of the possible effects of the anomalous decrease in the mobility of the dye-labeled lipid with concentration is self-aggregation, since Rhodamine is capable to form stable π-stacking self-interactions owing to its extended aromatic moiety. Indeed, Rhodamine was reported to form oligomers of growing size with concentration^32,33^, when dissolved in solution. We set out to demonstrate whether RHB aggregation occurred in the lipid membrane by recording the fluorescence spectra of RHB-DOPC GUVs at different concentration of the dye-labeled lipid. Figure 3f reports the fluorescence spectra for 0.1% and 10% RHB concentrations. The observed red-shift (about 8 nm) in the emission spectrum of the sample at high RHB concentration (10%) was interpreted as a fingerprint of the occurring aggregation of the fluorophore, also following previous detailed analyses^32,33^. On the other hand, no significant change in emission was observed at low concentration (<0.1%).

**Figure 4:**
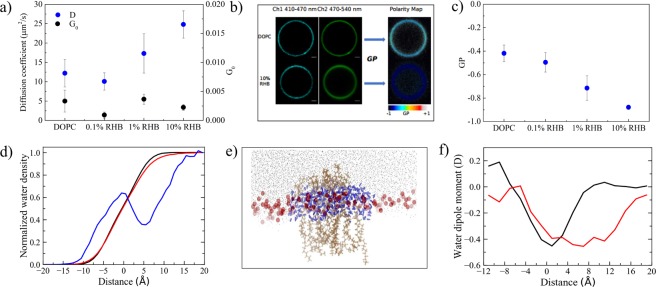
(**a**) Diffusion coefficient of LAURDAN increases with increasing concentration of RHB (blue dots), G_0_ remains constant (black dots). (**b**) Confocal microscopy images of GUVs labelled with LAURDAN, the emission signal was split in two channels and polarity maps were obtained from these images using the GP Equation (Eq. 1). (**c**) GP values obtained in GUVs at different concentration of RHB. Note that error bar is negligible at 10%. (**d**) Water density distribution evaluated along a direction normal to the membrane surface as issuing from MD simulations of DOPC (black), RHB-DOPC (red) and RHB^Agg^-DOPC (blue, water within 5 Å in XY plane from the center of the RHB cluster) lipid bilayers. Origin was set to the average position of P atoms in one leaflet, negative values correspond to the inner bilayer region, positive values to the outer region and bulk solution. Water density was normalized with respect to bulk density. (**e**) MD snapshot of the RHB cluster: RHB lipids are shown in orange, DOPC phosphate groups in red, water molecules within 5 Å from the RHB aggregate are in blue, while remaining water molecules at the membrane-water interface are in gray. (**f**) Average water dipole moment evaluated along a direction normal to the membrane surface as issuing from MD simulations of DOPC (black) and RHB^Agg^-DOPC (red, water within 5 Å in XY plane from the center of the RHB cluster) lipid bilayers.

Based on this observation, we decided to further investigate the properties of the mixed RHB-DOPC membrane with a second molecular probe, i.e. LAURDAN. LAURDAN is a membrane fluorescent dye which is known to integrate easily within the lipid bilayer owing to its highly hydrophobic tail. Due to its peculiarities, this optical probe is especially well suited to report on lipid structural changes or rearrangements. Moreover, LAURDAN is sensitive to the polarity of its micro-environment and displays a typical red shift in its emission spectrum upon change in dipolar relaxation caused by the increase of water content in the membrane^34,35^. First, we performed FCS measurements of LAURDAN diffusion coefficient when the probe was introduced in the same RHB-DOPC GUVs described above. In contrast with RHB, the diffusion coefficient of LAURDAN was observed to increase at higher RHB:DOPC concentration ratio (Table 2, Fig. 4a), reaching a maximum at 10% RHB in the considered concentration range. Then, in order to quantify any change in the spectral behavior of LAURDAN, the Generalized Polarization (GP) ratio of its emission spectrum was evaluated from GUV images according to Eq. 1 (see Methods section for details). In the polarity maps depicted in Fig. 4b different colors represent different GP values, according to the reported color scale. Indeed, low GP values indicated an enhanced water permeation into the membrane. As shown in Fig. 4c, LAURDAN GP values decreased with the increase of RHB concentration, thus supporting a higher water permeability of the RHB-DOPC lipid bilayer under such conditions.

### Modeling RHB aggregation: structural effects and lipid diffusion

A model of RHB lipid aggregation (i.e., RHB^Agg^-DOPC), consisting of 8 RHB molecules in the same layer of a DOPC membrane, was generated according to the computational protocol described in the Methods, see a molecular representation of the system in Fig. 1d,e and Supplementary Fig. S3. First, we analyzed some structural properties of the aggregated system. The P-P RDF of the RHB^Agg^-DOPC system has shown some deviations with respect to non-aggregated RHB-DOPC and pure DOPC systems (Fig. 2b): the structural perturbations caused by RHB lipids in the aggregated form, in the membrane upper layer, has clearly displayed a more structured profile than both the other systems. This is also a manifestation of the stability of the RHB assembly, which is maintained throughout the simulation with minor changes in the overall structure. In terms of deuterium order parameters, *S*_CD_, RHB lipids in the RHB^Agg^-DOPC system have shown a slightly higher ordered structure in comparison to non-aggregated systems (Fig. 2c,d). Moreover, to investigate possible structural changes of DOPC lipids in close proximity to the RHB assembly, *S*_CD_ was evaluated by considering only a selected region of DOPC lipids within 5 Å of any RHB molecule in the upper layer. Results have shown a slightly less ordered structure of DOPC in this region as compared to all other remaining DOPC molecules of the same RHB^Agg^-DOPC system (data not shown) and also to the pure DOPC system (Fig. 2e,f). Note that by extending the selection of DOPC lipids to a distance of 7.5 Å, i.e. including the whole first lipid shell around the RHB assembly, very similar results were obtained (see Supplementary Fig. S4). In the case of the aggregated system, the diffusion coefficient of the clustered RHB molecules was significantly lower, 4.0 ± 1.2 μm^2^ s^−1^, in comparison to the non-aggregated RHB systems, while surrounding DOPC lipids showed same mobility as in the pure DOPC system, 8.7 ± 1.0 μm^2^ s^−1^ (Table 1). The relative slower diffusion of RHB in the upper layer of the aggregated system was the consequence of the prolonged retention of the RHB molecules within the cluster throughout the simulation.

### Modeling RHB aggregation: water permeation into the membrane

**Figure 5:**
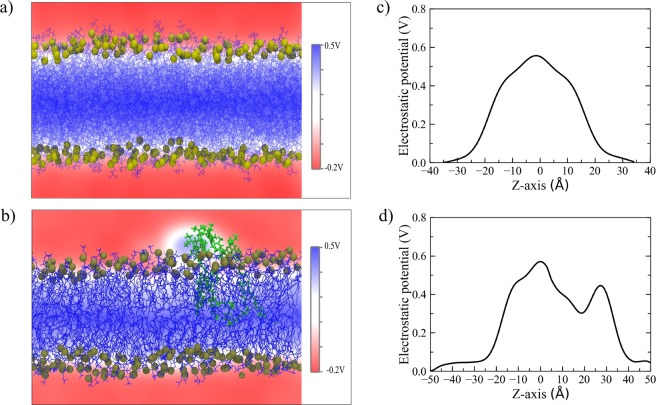
2D electrostatic potential maps as issuing from MD simulations of (**a**) DOPC and (**b**) RHB^Agg^-DOPC lipid bilayers evaluated on a lateral plane (i.e, XZ) of the simulation cell. Electrostatic potential evaluated along a direction perpendicular to the membrane surface as issuing from MD simulations of (**c**) DOPC and (**d**) RHB^Agg^-DOPC lipid bilayers. In the latter case, the normal was selected to pass through the center of the RHB cluster. Origin was set to the center of the bilayer in both cases. Overall, the electrostatic potential can change by up to 0.6 V across the lipid bilayer, but RHB^Agg^-DOPC system shows a high degree of asymmetry with a peak at about 30 Å in correspondence to the RHB aggregate.

We have analyzed the local water density going from bulk solution to the center of the lipid bilayer, along a direction normal to the membrane surface. Local water density was evaluated considering the position of water oxygen atoms in a range of distance between +20 Å and −20 Å with respect to the layer surface (i.e., average P coordinates). In the case of the RHB aggregated system, we applied a further restriction by selecting only the water molecules (i.e., oxygen atoms) falling within a distance of 5 Å from the center of the RHB assembly in the XY plane parallel to the membrane surface, thus analyzing the local water distribution in proximity of the dye assembly. Finally, water density was normalized with respect to bulk density, as depicted in Fig. 4d. In pure DOPC, the observed water density has shown a smooth drop while going from bulk (i.e., positive distance) towards the bilayer center (i.e., negative distance). A very similar density profile was observed in all considered RHB-DOPC systems. On the other hand, the water density profile displayed a peculiar non-monotonic trend when evaluated in the surrounding of the RHB aggregate from the MD simulation of the RHB^Agg^-DOPC bilayer, showing a marked increase (>25%) in water content inside the lipid bilayer in the region between 0 and −10 Å (as depicted in Fig. 4e). A peak in the water density was observed around the phosphate groups of the RHB lipids. This result clearly supported an increased water permeability of the lipid membrane in correspondence of the aggregate. The effects of the latter contributed also to a change in the mean dipole moment of water across the membrane surface as compared to the unlabeled lipid bilayer, as shown in Fig. 4f. The water dipole orientation is more pronounced in the headgroup region of the RHB aggregated system, possibly as a result of an enhanced local electric field due to the net electric charge on the RHB lipid and a decreased capability to screen effectively electrostatic interactions by the Rhodamine B moiety. Such an interfacial region was further investigated in both DOPC and RHB aggregated systems by evaluating the local electrostatic potential. Figure 5a–d reports the 2D electrostatic potential maps evaluated on a lateral plane of the lipid bilayer and the corresponding electrostatic potential along a normal to the membrane surface. Figure 5d highlights the asymmetric electrostatic potential in correspondence of the RHB cluster (i.e., peak around Z = 30 Å). We expect the qualitative picture emerging from Fig. 5d not to change significantly with cluster size since it refers to a local property (center of the cluster), at least for an extended concentration range not leading to lipid phase changes or other drastic phenomena.

## Discussion

In this work, we studied the structural, dynamic and self-aggregation properties of a dye-labeled lipid embedded in a simple DOPC planar membrane as a function of its concentration. Our combined MD-FCS approach provided new insights into the subtle interplay between dye concentration, lipid lateral diffusion and membrane permeability. On one hand, our study better assessed the role and the effects of a typical optical probe, here a Rhodamine B dye, if used to gather information on lipid dynamics from time-resolved measurements. In particular, we observed both a direct, concentration-independent, and an indirect, concentration-dependent, effect of the dye. Once tagged to a phospholipid, the tracer molecule induced a noticeable decrease (about 20%) in lipid lateral diffusion with respect to the unlabeled lipid, as estimated by our MD simulations (i.e., DOPC: *D* = 8.4 ± 0.4 μm^2^ s^−1^; RHB: *D = *6.7 ± 0.7 μm^2^ s^−1^). This is a direct manifestation of the higher hydrodynamic drag due to the bulky Rhodamine headgroup. Moreover, the diffusion coefficient obtained from FCS measurements on GUVs made up by a similar RHB-DOPC composition (i.e., 1%) with respect to our model displayed a somewhat higher but comparable value, 8.7 ± 2.4 μm^2^ s^−1^. This is a notable result considering the approximations affecting the model (e.g., forcefield parameters, periodic boundary conditions, planar lipid bilayer, etc.).

Conversely, FCS experiments at very dilute concentrations (10^−4^%) provided a diffusion coefficient of about 12 μm^2^ s^−1^. Since optical experiments can only probe the dynamics of dye-labeled lipids and assuming a dye hydrodynamic effect of 20%, our best estimate for lipid diffusion in pure DOPC membrane under normal conditions would be about 14 μm^2^ s^−1^. This result is within the range of previous studies on similar phospholipids (5–14 μm^2^ s^−1^)^6,9^, and it is close to the upper limit. Owing to the paramount importance of lipid mobility for setting the dynamics of complex biophysical and biochemical processes occurring at cell membranes, we believe that an accurate assessment of lipid lateral diffusion could be beneficial for a quantitative understanding of such phenomena. Besides, an accurate diffusion coefficient can serve as a useful benchmark for testing and developing new forcefields on which MD simulations of lipid bilayer rely upon. In this regard, we note that in our simulations DOPC diffusion (8.4 ± 0.4 μm^2^ s^−1^) seems a little underestimated with respect to the above “corrected” measure, a result possibly reflecting inaccuracies of the present model parameters. Moreover, the Saffman-Delbrück theory for periodic bilayers^19,20^ predicts our computed diffusion constant to be underestimated owing to the limited system size, while the accurate theoretical estimate can be recovered, in principle, at infinite size limit (for the present DOPC simulation, the predicted D^∞^ is ~22 μm^2^ s^−1^). Nevertheless, the above considerations on the hydrodynamic effect of the dye are reasonably not affected by the choice of periodic boundary conditions as they stem from a comparative study of same-size molecular systems.

Furthermore, our study reported a sensible slowdown of the diffusion mobility of the dye-labeled lipid in the concentration range going from 10^−4^% to 10%. Lipid lateral diffusion decreased from about 12 μm^2^ s^−1^ to 7 μm^2^ s^−1^, thus suggesting again a non-neutral role of the dye. Observed fluorescence spectra of GUVs recorded at low and high RHB concentration confirmed the occurrence of self-aggregation among dye-labeled lipids. This result was not surprising owing to the well-known capability of Rhodamine derivatives to undergo self-assembly in solution^32,33^. Nevertheless, our study clearly highlighted the impact of such transient lipid clusters on the resulting diffusive dynamics of the same dye-labeled lipids. Indeed, the increased inertial mass and hydrodynamic drag of such aggregates have a non-negligible effect on the observed lipid dynamics. While the extent of such an effect may depend on the peculiar chemical nature of the chosen tracer molecule, a similar behavior is expected to be shared by a large number of organic fluorophores typically employed as tracking probes in bioimaging studies. Note that self-aggregation could be perhaps one of the causes for the observed variability in lipid diffusion measurements recently reported by Guo *et al*.^15^ in lipid bilayers with different dye-labeled lipid content.

To proceed further, the observed self-aggregation was further investigated to highlight possible structural rearrangements of the membrane bilayer. The concomitant use of a second probe, LAURDAN, unraveled a pronounced change in membrane fluidity and water permeability upon formation of RHB aggregates (Fig. 4a–c). More insights emerged from our complementary modeling study of the RHB lipid self-assembly: the structural lipid-lipid rearrangements displayed by the computed P-P RDF (Fig. 2b) and the substantial change in the local electrostatic potential at the water-membrane interface (Fig. 5a–d) had the combined effect of enhancing water permeation into the interior of the membrane lipid bilayer, in stark contrast to the low concentration RHB-DOPC systems (Fig. 4d). Furthermore, the depletion in the water density in correspondence to the Rhodamine B headgroups (around 5 Å, Fig. 4d) reflects the hydrophobicity of the dye, an effect that may help to retain water molecules once inside the lipid bilayer. It is worth noting that no significant changes in lipid membrane structure was observed in our MD simulations of RHB-DOPC systems in absence of dye-labeled lipid cluster formation (Table 1 and Fig. 2). Hence, we think that pronounced structural changes in the present two-component dye-labeled/unlabeled lipid system can be mainly ascribed to the formation of such self-aggregates, and to a minor extent to the concentration ratio per se. Following self-aggregation, membrane structural changes are further boosted by the increase in water permeability. The increase in LAURDAN diffusivity with RHB concentration and the change in its photophysical response seem to agree nicely with the present interpretation.

Once more, our study confirms that lipid lateral diffusion is not only one of the most important dynamical parameters, but it is also intimately related to the membrane structure. Overall, the analyses carried out in the present study showed a non-neutral role of the tracer molecule in determining the lateral dynamics of the dye-labeled lipid as compared to the unlabeled parent lipid. The effect of the tagged dye becomes even more dramatic by increasing its concentration (>1%), since it enables the formation of self-aggregates and, in turn, a significant change in the local electric and structural properties of the membrane, also enhancing water permeability and fluidity at the same time.

While the main goal of this work was to develop an effective integrated approach based on FCS and MD analyses to investigate the qualitative and quantitative effects of the use of optical probes for tracking lipid lateral dynamics, our results pave the route to systematic studies on additional tracer/lipid systems under variable experimental conditions. We predict that a similar approach could be further extended to study multi-component lipid bilayers or protein mobility in membranes. A key point in our successful strategy is represented by the use of two optical probes to report concomitantly on the structural and dynamic properties of the lipid bilayer, respectively. While the use of multiple probes in fluorescence applications is, in general, a very attractive and fruitful approach, we would like to point out that extreme care has to be taken in the present context. First, one has to assess the specific choice of the fluorophores to avoid possible interferences in the optical signal, such as FRET. Then, as our study has clearly shown, one has to assess carefully whether unwanted effects are introduced by the probe itself with respect to the biophysical property under scrutiny. We believe that experimental results may inevitably suffer from the effect of lipid/protein labeling, to some extent. Further advances are expected by new generation of functional probes that report on membrane dynamical changes, and ultimately on the organizational hierarchy of cellular membranes, with negligible effects on the host systems.

## Methods

### Experimental protocol

The lipid DOPC, 1,2-dioleoyl-sn-glycero-3-phosphocholine (10 mg/mL in chloroform), and RHB, 1,2-dioleoyl-sn-glycero-3-phosphoethanolamine-N-(lissamine Rhodamine B sulfonyl) (ammonium salt), were purchased from Avanti Polar Lipids (Alabaster, AL, USA). LAURDAN (6-Dodecanoyl-2-Dimethylaminonaphthalene), low gelling temperature agarose, BioReagent, for molecular biology, and sucrose were purchased from Sigma Aldrich (St. Louis, MO, USA).

### Vesicles preparation and agarose embedding

Giant Unilamellar Vesicles (GUVs) of DOPC and at different concentrations of RHB (0.0001–0.1–1–10 mol%) were prepared by the electroformation method^36^. 5 µL of stock solution (1 mg/mL) in chloroform were spread on a pair of conductive ITO glass slide and dried for 5 minutes. CoverWell™ imaging chambers (Grace Bio-Labs) were used to create a chamber between the glasses, which was sealed and filled with a 0.3 M sucrose solution. The chamber was connected to a 3 V, 8 Hz AC current in order to induce formation of the vesicles at room temperature. An open source Arduino microcontroller was built and used to generate the AC field, analogously to what already proposed by Huynh *et al*.^37^. (see Supplementary Material for further details). After 7 hours, the frequency was changed to 4 Hz square wave for 1 h to detach the GUVs from glass. The content of the chambers was then collected and the electrodes were gently rinsed with the sucrose solution to remove any remaining vesicles. The GUVs suspension was then stored at 4 °C. LAURDAN was prediluted in DMSO (280 μM) and added at GUVs suspension. Agarose gel was used to immobilized GUVs as described in^38^. Agarose was dissolved in PBS at a concentration of 0.75% w/v. Liposomes were mixed in gel while the agarose was in the fluid state. After mixing, the solution was placed on a glass bottom petri dish and was left at room temperature for jellification.

### Data acquisition and analysis

FCS and spectra measurements were carried out on an Olympus FluoView FV-1000 inverted confocal microscope with a 60x water immersion objective (NA: 1.20). LAURDAN was imaged at 780 nm with two-photon excitation using a Titanium-Sapphire laser (Chameleon; Coherent, USA). The emission signal was collected in the 410–510 nm range. RHB was excited using a laser at 543 nm and the emission signal was collected in the range 550–650 nm. The emission spectrum of RHB was collected from 550 to 640 nm with a detection bandwidth of 2 nm and number of steps 42. The photomultipliers were set in the photon-counting detection mode. The Generalized Polarization (GP) measurement was performed on a Zeiss LSM 800 inverted confocal microscope equipped with two GaAsP detectors using a 63x oil immersion objective (NA 1.40). LAURDAN was excited at 405 nm. Then its emission was split by a dichroic mirror (with cutoff at 470 nm) into two detectors set to collect fluorescence in the 400–470 nm and 470–540 nm range, respectively. The emission spectrum of LAURDAN partially overlaps with the adsorption spectrum of Rhodamine B and Fluorescence Resonance Energy Transfer (FRET) may occur. In order to suppress FRET contribution to the measured GP, the acceptor Rhodamine B was photobleached before LAURDAN measurements. FCS measurements were analyzed with simFCS software (www.lfd.uci.edu, University of California Irvine). GP was calculated from the emission of the two channels using the following equation^34^:

1 $${\rm{GP}}=\frac{{I}_{440}-{I}_{490}}{{I}_{440}+{I}_{490}}$$

where *I*_440_ and *I*_490_ are the emission intensities at those wavelengths.

### Molecular models of DOPC and dye-labeled lipid

Starting structure for pure DOPC membrane bilayer was obtained from a pre-equilibrated system, using CHARMM-GUI server^39^. The bilayer consisted of 400 lipids (200 per leaflet) and extended up to ~125 Å along its lateral dimensions (XY plane). The system was then solvated with TIP3P water up to 30 Å from the bilayer along the normal to the membrane surface (Z-axis). Rhodamine B structure was initially generated with the Chimera software^40^. The starting geometry was optimized by quantum mechanical calculations using the B3LYP/6–31+G(d,p) level of theory using Gaussian09^41^, while partial charges were derived from molecular electrostatic potential obtained using the HF/6–31 G(d) level of theory in vacuum. The latter generally provides effectively “polarized” charges more suitable for modeling complex systems in the condensed phase without resorting on computationally demanding calculations of molecular polarization^42^. Note, also, that small deviations were observed when partial charges were computed using either B3LYP or including solvent effects through the Polarizable Continuum Model^43^ (mean absolute error < 0.1). Besides, in the present work we have neglected the possible electronic rearrangement occurring in the excited state of the dye^44^, assuming that, once tagged to the lipid, its orientation within the bilayer and diffusive dynamics would not be particularly affected. Rhodamine B was then attached to the lipid according to Fig. 1a in order to build a RHB molecular model. Overall electric charge of the dye-labeled lipid is −1e, since the phosphate group of the lipid is not compensated by the Rhodamine B headgroup. Forcefield parameters for the so obtained dye-labeled lipid were adapted from CHARMM general force field (CGenFF)^45^ for a small molecule (RHB force field details are provided in Supplementary Table S3). On the other hand, RHB lipid tail parameters were borrowed from the corresponding standard DOPC CHARMM parameters, being the two structures chemically equivalent. Eight RHB lipid molecules (4 per leaflet) were then embedded in a well-equilibrated pure DOPC bilayer, previously created as described above, by replacing a corresponding number of DOPC lipids at random locations. The final structure had 196 DOPC and 4 RHB molecules in each membrane leaflet. Water molecules in close contact with the RHB headgroup were deleted and an additional water layer (extending up to ~20 Å) was added along the Z-dimension to build up the final system (box edges: 117 × 117 × 92 Å^3^, water thickness: ~54 Å). Eight sodium ions were added to keep the system electrically neutral. The RHB-DOPC bilayer is depicted in Fig. 1b,c. Four different replicas of the RHB-DOPC system were produced by regenerating the velocities in each system replica. In addition, one more RHB-DOPC system was created from one of the above replicas with a salt concentration of 0.5 M NaCl. In all cases, force field included NBFIX terms for sodium in interaction with chloride ions and carboxylate groups^46,47^.

### Molecular model of an aggregated cluster of dye-labeled lipids

Moreover, we generated a molecular model of an aggregated cluster of RHB lipids within a DOPC bilayer (i.e., RHB^Agg^-DOPC). Initially, a well-equilibrated RHB-DOPC configuration was taken, and at the center of one leaflet (i.e., upper leaflet) eight RHB lipids were introduced in a stacked configuration, while keeping four RHB lipids in the other (lower) leaflet at random locations. DOPC lipids showing clash contacts with any RHB molecules were removed. The overall RHB aggregated system had: 8 RHB (forming a cluster) and 182 DOPC molecules in the upper layer, 4 RHB (randomly distributed) and 186 DOPC molecules in the lower layer (box edges: 114 × 114 × 105 Å^3^, water thickness: ~67 Å). Counterions were also added to neutralize the overall electric charge of the system. The initial system configuration was then relaxed following the simulation protocol described below. A molecular representation of the system is shown in Fig. 1d,e.

### Simulation details

All simulations were performed in NAMD 2.10^48^ using CHARMM36^49^ force field for lipids under periodic boundary condition. Bonds involving hydrogens were restrained using SHAKE^50^, which permitted the usage of a 2 fs time step for numerical integration. Long-range electrostatic interactions were calculated using the Particle Mesh Ewald (PME) algorithm^51^, while dispersion interactions were calculated with a cutoff of 12 Å, applying a smoothing functions beyond 10 Å. All production MD simulations were performed in a NPT ensemble with a constant pressure of 1 atm and a temperature of 303 K, using a Langevin thermostat and barostat for pressure and temperature coupling, respectively^52^. All simulations were performed with a cell box constant-ratio constraint, which allows to keep the ratio of the unit cell constant in X and Y axis while allows fluctuation along all three dimensions. Prior to production phase, all systems were subject to a short round of energy minimization and system density equilibration (1000 steps of steepest-descent minimization and about 50 ns of MD equilibration). After equilibration, all MD simulations were carried out for about 200 ns, except the four RHB-DOPC replica system, for which we collected about 100 ns each. For the aggregated RHB system, an initial NpT simulation was performed while applying harmonic position restraints on the headgroups of RHB lipids for about 30 ns. In order to speed up the aggregation process and create a RHB cluster not biased towards a specific configuration, we applied a harmonic restraint to the radius of gyration (Rg) of the eight Rhodamine B headgroups in the upper layer, using the collective variable module of NAMD (force constant 1 kcal/mol Å^−1^, Rg 1.0 nm), thus obtaining an equilibrated RHB cluster as shown in Fig. 1d,e.

### Data Analysis

Dynamics of the pure (DOPC) and modified (RHB) lipid bilayers have been characterized via a number of properties such as (i) area per lipid (ApL), (ii) membrane thickness, (iii) radial distribution function (RDF), (iv) diffusion coefficient (D), and (v) lipid order parameters (S_CD_), using in-house developed codes based on the MDAnalysis library^53^ or available analysis tools in Gromacs^54^ and Amber^54,55^ software. Average area per lipid (ApL) was obtained from the average area of the membrane surface (i.e., XY plane) divided by the total number of lipids in each layer. Membrane thickness was obtained from the average distance between the phosphate groups belonging to the upper and lower leaflets. Radial distribution functions (RDF) were evaluated by considering the interatomic distances of the P atoms belonging to the phosphate groups of either DOPC or RHB, performing distinct analyses on each leaflet and then averaging the results. Diffusion coefficient for lateral lipid diffusion was obtained from the mean square displacement (MSD) of the phosphate atoms, removing the effect of the center of mass motion of the entire system, using the Einstein relation:

2 $${\rm{MSD}}(t)=4Dt$$

and performing a linear fitting of the MSD data on the time interval between 11 and 50 ns. Deuterium order parameter (S_CD_) was evaluated by considering the average orientation of the methylene groups along the lipid hydrocarbon chains with respect to the membrane normal^56^, and computed as:

3 $${S}_{{\rm{CD}}}=\langle \frac{1}{2}(3{\cos }^{2}{{\rm{\Theta }}}_{{\rm{CD}}}-1)\rangle $$

where Θ_*CD*_ is the angle between each CH bond and the normal to the membrane (Z-axis), and angular brackets denote an average over all lipids and ensemble configurations. Uncertainties in the estimates of the above properties were evaluated as standard errors from multiple partitions (i.e.,^8^) of the simulated trajectories. In case of RHB-DOPC simulations, standard errors were computed by considering separately the upper and lower leaflets in all replica simulations.

## Supplementary information

Supplementary Material

## Data Availability

All data presented in the present work is fully available upon request.
