# Supplementary material for: Interplay between lipid lateral diffusion, dye concentration and membrane permeability unveiled by a combined spectroscopic and computational study of a model lipid bilayer
Source: Sci Rep. 2019 Feb 6;9:1508. doi: 10.1038/s41598-018-37814-x (PMC6365552; doi:10.1038/s41598-018-37814-x)
Supplement: Supplementary file 1 — Supplementary Material [file 41598_2018_37814_MOESM1_ESM.pdf]

Supplementary Material for

Interplay between lipid lateral diffusion, dye concentration and membrane permeability unveiled by a combined spectroscopic and computational study of a model lipid bilayer

Muhammad Jan Akhunzada,[a,b+] Francesca D'Autilia,[c+] Balasubramanian Chandramouli,[a,#] Nicholus Bhattacharjee,[a,b] Andrea Catte,[a,b] Roberto Di Rienzo, [d] Francesco Cardarelli,[e] and Giuseppe Brancato[a,b*]

[a] Scuola Normale Superiore, piazza dei Cavalieri 7, I-56126 Pisa, Italy.

[b] Istituto Nazionale di Fisica Nucleare, Largo Pontecorvo 3, I-56100 Pisa, Italy

[c] Center for Nanotechnology Innovation@NEST (CNI@NEST), Pisa, Italy

[d] Dipartimento di Ingegneria dell'Informazione, Università di Pisa, Italy

[e] NEST, Scuola Normale Superiore and Istituto Nanoscienze-CNR, Piazza San Silvestro 12 - 56127 Pisa, Italy
# Present address: Compunet, Istituto Italiano di Tecnologia (IIT), Via Morego 30, I-16163 Genova, Italy

*Corresponding author:

E-mail: giuseppe.brancato@sns.it

Content

1) **Figure S1**. Minimum RHB-RHB distance, as a function of time.

2) **Figure S2**. P-Na^+ radial distribution functions from RHB-DOPC simulation.

3) **Table S1**. Statistical T-test for the RHB diffusion values reported in Table 2.

4) **Table S2**. Statistical T-test for the LAURDAN diffusion values reported in Table 2.

5) **Figure S3**. Side view of the eight aggregated RHB lipids.

6) **Figure S4.** Deuterium order parameters (S_{CD}) evaluated for DOPC acyl chains.

7) **Table S3**. CHARMM model for the RHB lipid, reporting atom types and atomic charges.

8) Circuit diagram and Arduino code used for electroformation.

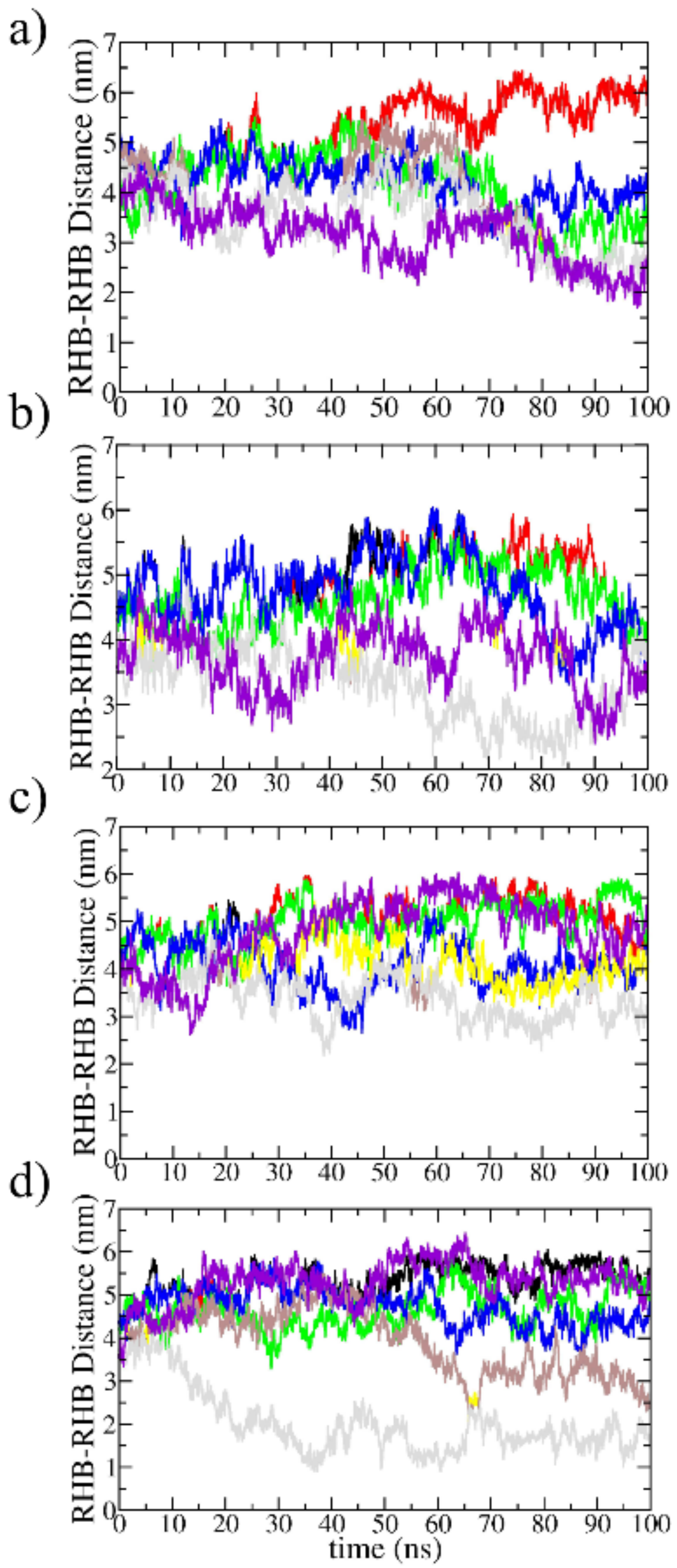

Figure S1. Minimum RHB-RHB distance, as a function of time, between each RHB phosphate group (i.e., P atom) and all the others in both upper and lower leaflet as issuing from all four RHB-DOPC MD simulations (a-d). Each colored line refers to a different RHB lipid. RHB-RHB distances are on average ~40 Å. No RHB lipid is observed to approach the first shell of any other one.

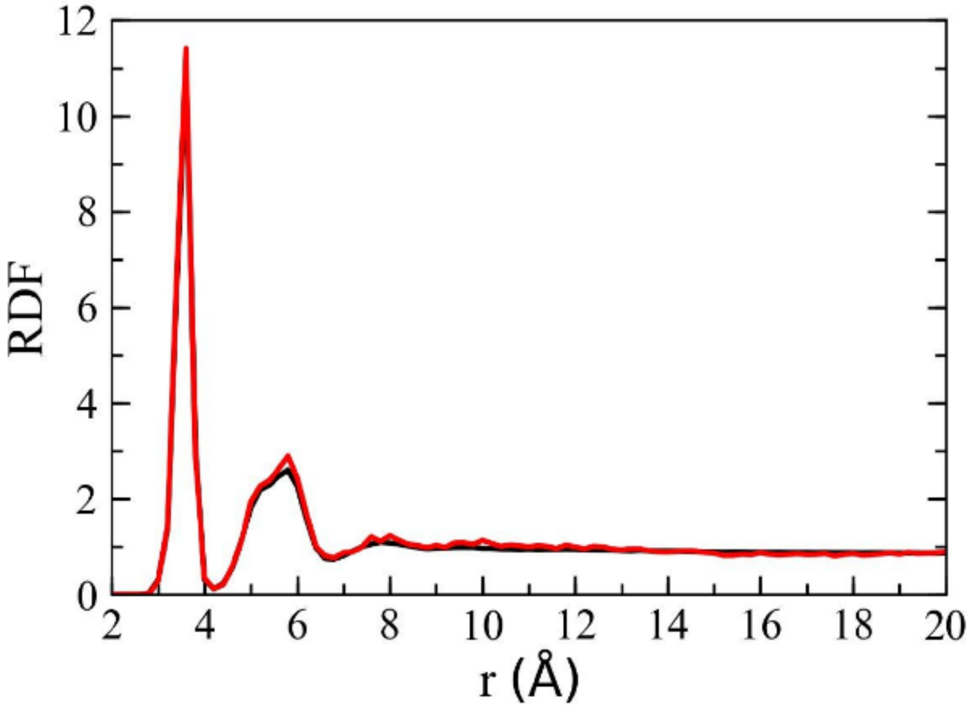

Figure S2. P - Na^+ radial distribution functions (RDFs) obtained considering DOPC (black line) and RHB (red line) lipids as issuing from the RHB-DOPC MD simulation with 0.5M NaCl. Sodium ions are observed to penetrate the phosphate region of the lipid bilayer in a similar way with respect to both RHB and DOPC lipids.

Table S1. Statistical t-test for the RHB diffusion values reported in Table 2.

	**0.0001% RHB**	**0.1% RHB**	**1% RHB**	**10% RHB**
**0.0001% RHB**	N.D.	0.1921*	0.0009	<0.0001
**0.1% RHB**	-	N.D.	0.015	<0.0001
**1% RHB**	-	-	N.D.	0.0337
**10% RHB**	-	-	-	N.D.

*Not statistically different.

Table S2. Statistical t-test for the LAURDAN diffusion values reported in Table 2.

	**DOPC**	**0.1% RHB**	**1% RHB**	**10% RHB**
**DOPC**	N.D.	0.1585*	0.0229	0.0003
**0.1% RHB**	-	N.D.	0.0007	<0.0001
**1% RHB**	-	-	N.D.	0.0215
**10% RHB**	-	-	-	N.D.

*Not statistically different.

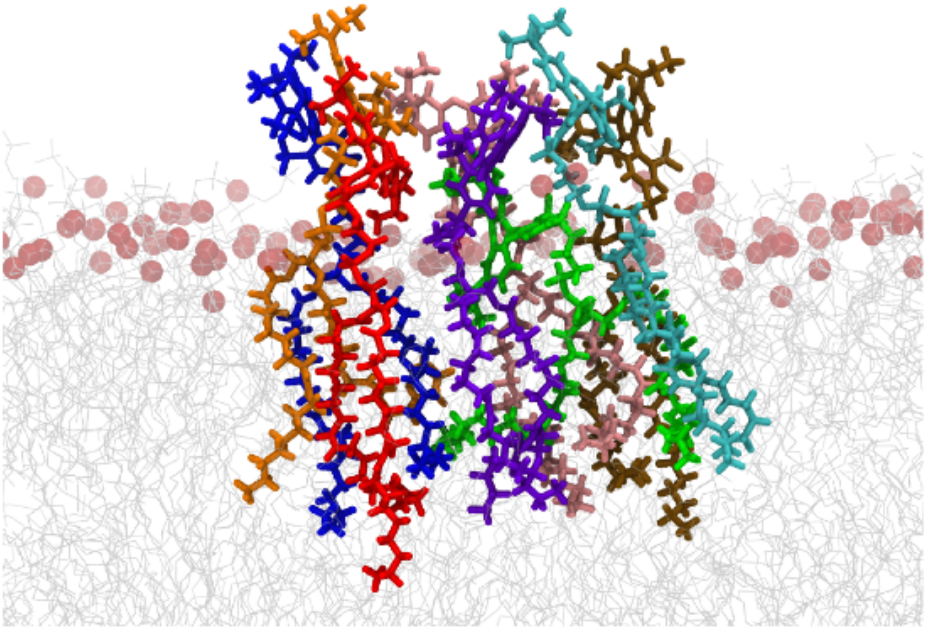

Figure S3. Side view of the eight aggregated RHB lipids, each shown with a different color, within the DOPC bilayer. DOPC lipids are depicted in gray with phosphorus atom represented as red beads.

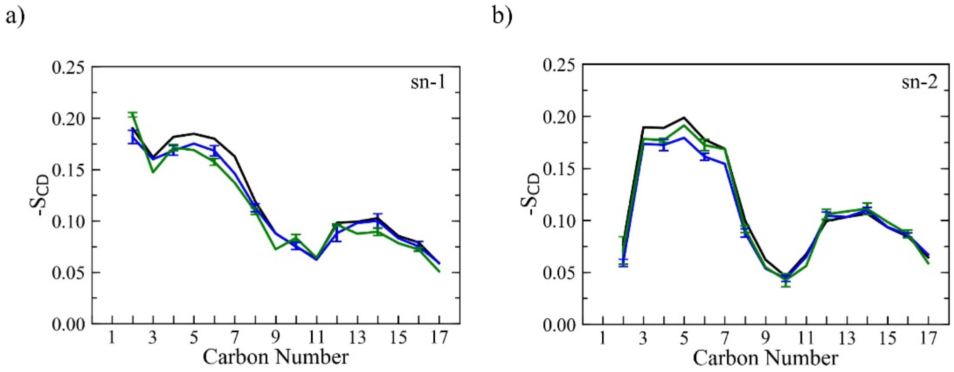

Figure S4. a,b) Deuterium order parameters (S_{CD}) evaluated for lipid acyl chains (i.e., sn-1 and sn-2) as issuing from MD simulations of DOPC (black, DOPC lipids), RHB^{Agg}-DOPC (blue, only DOPC lipids within 5 Å from any RHB lipid) and RHB^{Agg}-DOPC (green, only DOPC lipids within 7.5 Å from any RHB lipid) lipid bilayers. Small differences in DOPC order parameters appeared as caused by the local interaction with RHB lipids. In all diagrams, error bars correspond to one standard errors. Error bars of pure DOPC system (black lines) are negligible and then omitted for clarity in all plots.

Table S3. List of atoms of the RHB lipid model reporting atom types and atomic charges.

```
* -------------------------------------------------------------------------------------- *
*       CGenFF: Topology for the Charmm General Force Field v. 2b7        *
*               for Small Molecule Drug Design                              *
* -------------------------------------------------------------------------------------- *
*
36  1
! -------------------------------------------------------------------------------------- !


RESI RHB
GROUP

ATOM S1f      SG3O2     1.100
ATOM O1f      OG2P1    -0.570
ATOM O2f      OG2P1    -0.570
ATOM C1f      CG2R61   -0.150
ATOM C2f      CG2R61    0.020
ATOM H3f      HGR61     0.150
ATOM C3f      CG2R61   -0.290
ATOM H4f      HGR61     0.090
ATOM C4f      CG2R67    0.310
ATOM C5f      CG2R61   -0.140
ATOM C6f      CG2R61   -0.060
ATOM H5f      HGR61     0.150
ATOM S2f      SG3O1     1.100
ATOM O3f      OG2P1    -0.700
ATOM O4f      OG2P1    -0.700
ATOM O5f      OG2P1    -0.700
ATOM C7f      CG2R67    0.330
ATOM C8f      CG2R61   -0.300
ATOM C9f      CG2R61    0.610
ATOM O6f      OG3R60   -0.430
ATOM C10f     CG2R61    0.610
ATOM C11f     CG2R61   -0.300
ATOM C12f     CG2DC1    0.160
ATOM H6f      HGA4      0.08
ATOM C13f     CG2DC1   -0.460
ATOM H7f      HGA4      0.210
ATOM C14f     CG2DC2    0.640
ATOM C15f     CG2DC1   -0.640
ATOM H8f      HGA4      0.250
ATOM N2f      NG2P1    -0.50
ATOM C16f     CG324     0.23
ATOM H9f      HGA2      0.01
ATOM H10f     HGA2      0.01
ATOM C17f     CG331    -0.270
ATOM H11f     HGA3      0.090
ATOM H12f     HGA3      0.090
ATOM H13f     HGA3      0.090
ATOM C18f     CG324     0.23
ATOM H14f     HGA2      0.01
ATOM H15f     HGA2      0.01
ATOM C19f     CG331    -0.270
ATOM H16f     HGA3      0.090
ATOM H17f     HGA3      0.090
ATOM H18f     HGA3      0.090
ATOM C20f     CG2R61    0.160
ATOM H19f     HGR61     0.08
ATOM C21f     CG2R61   -0.460
ATOM H20f     HGR61     0.210
```

```
ATOM C22f        CG2R61      0.640
ATOM C23f        CG2R61     -0.640
ATOM H21f        HGR61       0.250
ATOM N3f         NG301      -0.50
ATOM C24f        CG321       0.23
ATOM H22f        HGA2        0.01
ATOM H23f        HGA2        0.01
ATOM C25f        CG331      -0.270
ATOM H24f        HGA3        0.090
ATOM H25f        HGA3        0.090
ATOM H26f        HGA3        0.090
ATOM C26f        CG321       0.23
ATOM H27f        HGA2        0.01
ATOM H28f        HGA2        0.01
ATOM C27f        CG331      -0.270
ATOM H29f        HGA3        0.090
ATOM H30f        HGA3        0.090
ATOM H31f        HGA3        0.090


! Polar Head and glycerol backbone
GROUP             !

ATOM N    NH3L  -0.670 !          HN2
!ATOM HN1  HCL    0.33 !           |
!ATOM HN2  HCL    0.33 !  (+) HN1---N---HN3
ATOM HN3  HCL    0.350 !           |
ATOM C12  CTL2   0.100 !           |
ATOM H12A HAL2   0.090 !    H12A--C12---H12B
ATOM H12B HAL2   0.090 !           |
GROUP             !                |    alpha5
ATOM C11  CTL2  -0.08 !            |
ATOM H11A HAL2   0.09 !    H11A--C11---H11B
ATOM H11B HAL2   0.09 !            |    alpha4
ATOM P    PL     1.50 !  (-) O13  O12
ATOM O13  O2L   -0.78 !        \ /      alpha3
ATOM O14  O2L   -0.78 !         P (+)
ATOM O11  OSLP  -0.57 !        / \      alpha2
ATOM O12  OSLP  -0.57 !  (-) O14  O11
ATOM C1   CTL2  -0.08 !          |    alpha1
ATOM HA   HAL2   0.09 !      HA---C1---HB
ATOM HB   HAL2   0.09 !           |    theta1
GROUP             !               |
ATOM C2   CTL1   0.17 !      HS---C2- - - - - - -
ATOM HS   HAL1   0.09 !           | beta1       |
ATOM O21  OSL   -0.49 !      O22  O21         theta3
ATOM C21  CL     0.90 !        \\ /  beta2      |
ATOM O22  OBL   -0.63 !         C21
ATOM C22  CTL2  -0.22 !         |  beta3       |
ATOM H2R  HAL2   0.09 !  H2R---C22---H2S
ATOM H2S  HAL2   0.09 !         |              |
GROUP             !           beta4
ATOM C3   CTL2   0.08 !         |              |
ATOM HX   HAL2   0.09 !                   HX---C3---HY
ATOM HY   HAL2   0.09 !         |              |  gamma1
ATOM O31  OSL   -0.49 !                   O32  O31
ATOM C31  CL     0.90 !        |           \\ /   gamma2
ATOM O32  OBL   -0.63 !                     C31
ATOM C32  CTL2  -0.22 !         |           |    gamma3
ATOM H2X  HAL2   0.09 !               H2X---C32---H2Y
ATOM H2Y  HAL2   0.09 !         |           |
GROUP             !                   gamma4
ATOM C23  CTL2  -0.18 !         |           |
```

```
ATOM H3R  HAL2   0.09 !   H3R ---C23---H3S      |
ATOM H3S  HAL2   0.09 !          |            |
GROUP                 !         |            |
ATOM C24  CTL2  -0.18 !          |            |
ATOM H4R  HAL2   0.09 !   H4R ---C24---H4S      |
ATOM H4S  HAL2   0.09 !          |            |
GROUP                 !         |            |
ATOM C25  CTL2  -0.18 !          |            |
ATOM H5R  HAL2   0.09 !   H5R ---C25---H5S      |
ATOM H5S  HAL2   0.09 !          |            |
GROUP                 !         |            |
ATOM C26  CTL2  -0.18 !          |            |
ATOM H6R  HAL2   0.09 !   H6R ---C26---H6S      |
ATOM H6S  HAL2   0.09 !          |            |
GROUP                 !         |            |
ATOM C27  CTL2  -0.18 !          |            |
ATOM H7R  HAL2   0.09 !   H7R ---C27---H7S      |
ATOM H7S  HAL2   0.09 !          |            |
GROUP                 !         |            |
ATOM C28  CTL2  -0.18 !          |            |
ATOM H8R  HAL2   0.09 !   H8R ---C28---H8S      |
ATOM H8S  HAL2   0.09 !          |            |
GROUP                 !         |            |
ATOM C29  CEL1  -0.15 !          |            |
ATOM H9R  HEL1   0.15 !   H9R ---C29            |
GROUP                 !         ||  (CIS)      |
ATOM C210 CEL1  -0.15 !          ||           |
ATOM H10R HEL1   0.15 !   H10R---C210          |
GROUP                 !         |            |
ATOM C211 CTL2  -0.18 !          |            |
ATOM H11R HAL2   0.09 !   H11R---C211--H11S     |
ATOM H11S HAL2   0.09 !          |            |
GROUP                 !         |            |
ATOM C212 CTL2  -0.18 !          |            |
ATOM H12R HAL2   0.09 !   H12R---C212--H12S     |
ATOM H12S HAL2   0.09 !          |            |
GROUP                 !         |            |
ATOM C213 CTL2  -0.18 !          |            |
ATOM H13R HAL2   0.09 !   H13R---C213--H13S     |
ATOM H13S HAL2   0.09 !          |            |
GROUP                 !         |            |
ATOM C214 CTL2  -0.18 !          |            |
ATOM H14R HAL2   0.09 !   H14R---C214--H14S     |
ATOM H14S HAL2   0.09 !          |            |
GROUP                 !         |            |
ATOM C215 CTL2  -0.18 !          |            |
ATOM H15R HAL2   0.09 !   H15R---C215--H15S     |
ATOM H15S HAL2   0.09 !          |            |
GROUP                 !         |            |
ATOM C216 CTL2  -0.18 !          |            |
ATOM H16R HAL2   0.09 !   H16R---C216--H16S     |
ATOM H16S HAL2   0.09 !          |            |
GROUP                 !         |            |
ATOM C217 CTL2  -0.18 !          |            |
ATOM H17R HAL2   0.09 !   H17R---C217--H17S     |
ATOM H17S HAL2   0.09 !          |            |
GROUP                 !         |            |
ATOM C218 CTL3  -0.27 !          |            |
ATOM H18R HAL3   0.09 !   H18R---C218--H18S     |
ATOM H18S HAL3   0.09 !          |            |
ATOM H18T HAL3   0.09 !          H18T          |
GROUP                 !                      |
ATOM C33  CTL2  -0.18 !                       |
```

```
ATOM H3X  HAL2   0.09 !                 H3X ---C33---H3Y
ATOM H3Y  HAL2   0.09 !                         |
GROUP              !                  |
ATOM C34  CTL2  -0.18 !                        |
ATOM H4X  HAL2   0.09 !                 H4X ---C34---H4Y
ATOM H4Y  HAL2   0.09 !                         |
GROUP              !                  |
ATOM C35  CTL2  -0.18 !                        |
ATOM H5X  HAL2   0.09 !                 H5X ---C35---H5Y
ATOM H5Y  HAL2   0.09 !                         |
GROUP              !                  |
ATOM C36  CTL2  -0.18 !                        |
ATOM H6X  HAL2   0.09 !                 H6X ---C36---H6Y
ATOM H6Y  HAL2   0.09 !                         |
GROUP              !                  |
ATOM C37  CTL2  -0.18 !                        |
ATOM H7X  HAL2   0.09 !                 H7X ---C37---H7Y
ATOM H7Y  HAL2   0.09 !                         |
GROUP              !                  |
ATOM C38  CTL2  -0.18 !                        |
ATOM H8X  HAL2   0.09 !                 H8X ---C38---H8Y
ATOM H8Y  HAL2   0.09 !                         |
GROUP              !                  |
ATOM C39  CEL1  -0.15 !                        |
ATOM H9X  HEL1   0.15 !                 H9X ---C39
GROUP              !                  ||
ATOM C310 CEL1  -0.15 !                         ||
ATOM H10X HEL1   0.15 !                 H10X---C310
GROUP              !                  |
ATOM C311 CTL2  -0.18 !                        |
ATOM H11X HAL2   0.09 !                 H11X---C311--H11Y
ATOM H11Y HAL2   0.09 !                         |
GROUP              !                  |
ATOM C312 CTL2  -0.18 !                        |
ATOM H12X HAL2   0.09 !                 H12X---C312--H12Y
ATOM H12Y HAL2   0.09 !                         |
GROUP              !                  |
ATOM C313 CTL2  -0.18 !                        |
ATOM H13X HAL2   0.09 !                 H13X---C313--H13Y
ATOM H13Y HAL2   0.09 !                         |
GROUP              !                  |
ATOM C314 CTL2  -0.18 !                        |
ATOM H14X HAL2   0.09 !                 H14X---C314--H14Y
ATOM H14Y HAL2   0.09 !                         |
GROUP              !                  |
ATOM C315 CTL2  -0.18 !                        |
ATOM H15X HAL2   0.09 !                 H15X---C315--H15Y
ATOM H15Y HAL2   0.09 !                         |
GROUP              !                  |
ATOM C316 CTL2  -0.18 !                        |
ATOM H16X HAL2   0.09 !                 H16X---C316--H16Y
ATOM H16Y HAL2   0.09 !                         |
GROUP              !                  |
ATOM C317 CTL2  -0.18 !                        |
ATOM H17X HAL2   0.09 !                 H17X---C317--H17Y
ATOM H17Y HAL2   0.09 !                         |
GROUP              !                  |
ATOM C318 CTL3  -0.27 !                        |
ATOM H18X HAL3   0.09 !                 H18X---C318--H18Y
ATOM H18Y HAL3   0.09 !                         |
ATOM H18Z HAL3   0.09 !                        H18Z

! Bond order
```

BOND S1f O1f S1f O2f S1f C1f
BOND C1f C2f C1f C6f
BOND C2f H3f
BOND C2f C3f
BOND C3f H4f
BOND C3f C4f
BOND C4f C5f
BOND C4f C7f
BOND C5f C6f
BOND C5f S2f
BOND C6f H5f
BOND S2f O3f
BOND S2f O4f
BOND S2f O5f
BOND C7f C8f
BOND C7f C11f
BOND C8f C9f
BOND C8f C12f
BOND C9f O6f
BOND C9f C15f
BOND O6f C10f
BOND C10f C11f
BOND C10f C23f
BOND C11f C20f
BOND C12f H6f
BOND C12f C13f
BOND C13f H7f
BOND C13f C14f
BOND C14f C15f
BOND C14f N2f
BOND C15f H8f
BOND N2f C16f
BOND N2f C18f
BOND C16f H9f
BOND C16f H10f
BOND C16f C17f
BOND C17f H11f
BOND C17f H12f
BOND C17f H13f
BOND C18f H14f
BOND C18f H15f
BOND C18f C19f
BOND C19f H16f
BOND C19f H17f
BOND C19f H18f
BOND C20f H19f
BOND C20f C21f
BOND C21f H20f
BOND C21f C22f
BOND C22f C23f
BOND C22f N3f
BOND C23f H21f
BOND N3f C24f
BOND N3f C26f
BOND C24f H22f
BOND C24f H23f
BOND C24f C25f
BOND C25f H24f
BOND C25f H25f
BOND C25f H26f
BOND C26f H27f
BOND C26f H28f

```
BOND C26f C27f
BOND C27f H29f
BOND C27f H30f
BOND C27f H31f


! CONNECT FLUOROPHORE TO DOPE

BOND S1f   N

! Polar headgroup

BOND  N    HN3      N    C12
BOND  C12  H12A     C12  H12B     C12  C11
BOND  C11  H11A     C11  H11B     C11  O12
BOND  O12  P        P    O11      P    O13      P    O14

! Glycerol backbone

BOND  C1   HA       C1   HB       C1   C2       C1   O11
BOND  C2   HS       C2   C3       C2   O21
BOND  C3   HX       C3   HY       C3   O31

! Chain from C2

BOND  O21  C21
BOND  C21  C22
DOUBLE  C21  O22
BOND  C22  H2R      C22  H2S      C22  C23
BOND  C23  H3R      C23  H3S      C23  C24
BOND  C24  H4R      C24  H4S      C24  C25
BOND  C25  H5R      C25  H5S      C25  C26
BOND  C26  H6R      C26  H6S      C26  C27
BOND  C27  H7R      C27  H7S      C27  C28
BOND  C28  H8R      C28  H8S      C28  C29
BOND  C29  H9R
DOUBLE  C29 C210
BOND  C210 H10R     C210 C211
BOND  C211 H11R     C211 H11S     C211 C212
BOND  C212 H12R     C212 H12S     C212 C213
BOND  C213 H13R     C213 H13S     C213 C214
BOND  C214 H14R     C214 H14S     C214 C215
BOND  C215 H15R     C215 H15S     C215 C216
BOND  C216 H16R     C216 H16S     C216 C217
BOND  C217 H17R     C217 H17S     C217 C218
BOND  C218 H18R     C218 H18S     C218 H18T

! Chain from C3

BOND  O31  C31
BOND  C31  C32
DOUBLE  C31  O32
BOND  C32  H2X      C32  H2Y      C32  C33
BOND  C33  H3X      C33  H3Y      C33  C34
BOND  C34  H4X      C34  H4Y      C34  C35
BOND  C35  H5X      C35  H5Y      C35  C36
BOND  C36  H6X      C36  H6Y      C36  C37
BOND  C37  H7X      C37  H7Y      C37  C38
BOND  C38  H8X      C38  H8Y      C38  C39
BOND  C39  H9X
DOUBLE C39 C310
BOND  C310 H10X     C310 C311
BOND  C311 H11X     C311 H11Y     C311 C312
```

```
BOND  C312 H12X     C312 H12Y     C312 C313
BOND  C313 H13X     C313 H13Y     C313 C314
BOND  C314 H14X     C314 H14Y     C314 C315
BOND  C315 H15X     C315 H15Y     C315 C316
BOND  C316 H16X     C316 H16Y     C316 C317
BOND  C317 H17X     C317 H17Y     C317 C318
BOND  C318 H18X     C318 H18Y     C318 H18Z

IMPR C14f   C13f   C15f   N2f
IMPR C21 O21 C22 O22   C31 O31 C32 O32

END
```

Supporting Text

Circuit diagram and Arduino code used for electroformation

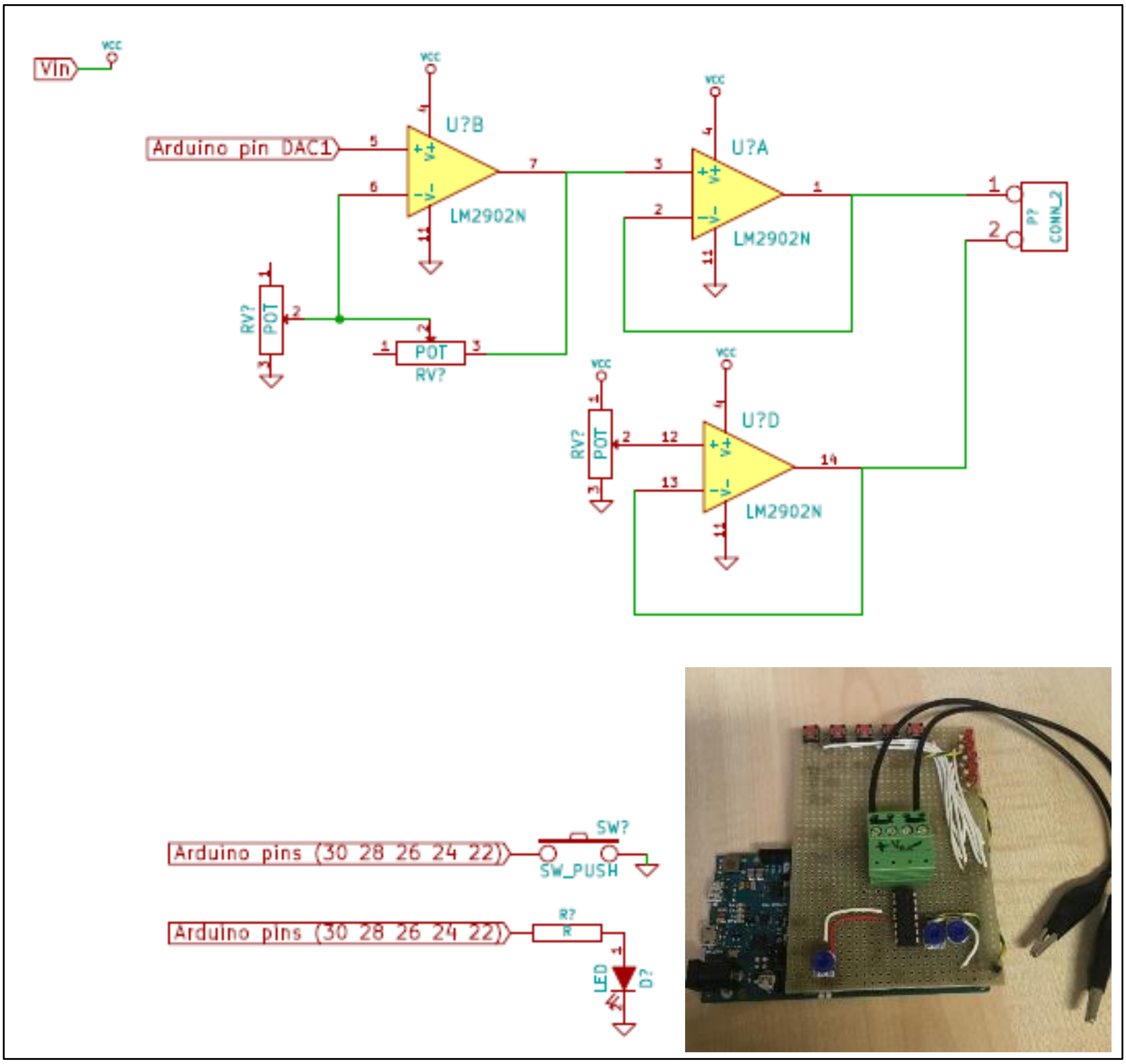

```
#define LR1 31
#define LR2 29
#define LR3 27
#define LR4 25
#define LR5 23
#define P1 30
#define P2 28
#define P3 26
#define P4 24
#define P5 22
#define OUT DAC1

// Variabili g1oba1i (tutti interi)
int countButton = 0; // Counter for the button
int IncreasingStep = 12; // Number of Steps in the starting
ramp
int IncreasingStepCounter = 1;
//int IncreasingTime = 30*60*1000; // Increasing Time in
mseconds
int IncreasingTime = 30*1000;
int IncreasingPeriod= 125; // Increasing Period in mseconds
long WorkingVoltage = 2500; // mVo1t peak to peak
int mVoltForBit = 1.953125;
int Vapplied;
int GrowthPeriod = 125; // Growth Period in mseconds
int DetachingStep = 8; // Number of Steps in the starting ramp
int DetachingStepCounter = 1;
int DetachingTime = 60*60*1000; // Increasing Time in mseconds
int DetachingPeriodIn = 100; // Increasing Period in mseconds
int DetachingPeriodFin = 10; // Increasing Period in mseconds
int DetachingPeriod;

//funzioni
void square (int v,int t)
{
  int n=(int)(v/0.001953125);
  analogWrite(OUT, 2048+n);
   delay( (int) (t/2) );
  analogWrite(OUT, 2048-n);
```

```
  delay((int)(t/2));
  analogWrite(OUT, 2048);
}

void quadra (unsigned long periodo,unsigned long tempo,int
n_max,int n_min){
  unsigned long n_periodi=tempo/periodo;
  for(unsigned long i=0;i<=n_periodi;i++){
    //semionda negetiva
    digitalWrite(LR1 ,LOW);
    analogWrite(OUT,n_min);
   delayMicroseconds(1000*periodo/2);
    //semionda positive
    digitalWrite(LR1 ,HIGH);
    analogWrite(OUT,n_max);
   delayMicroseconds(1000*periodo/2);
  }
}


void sinusoidale (unsigned long periodo,unsigned long tempo,
int n_max,int n_min)
{
  int Steps=200;
  int n_pwm;
  int aux=(n_max-n_min)/2;
  unsigned long n_periodi=tempo/periodo;
  for(unsigned long i=0;i<=n_periodi;i++)
  {
    for(float j=0; j<=(2*3.14); j=j+(2*3.14/Steps) )
     {
      //ein(red)=[-l,1]
      n_pwm= (sin(j)+1)*aux + n_min;
      analogWrite(OUT,n_pwm);
     delayMicroseconds(1000*periodo/Steps);
     }
  }
}

void triangolare (unsigned long periodo,unsigned long tempo,
int n_max,int n_min){
```

```
  int Steps=200;
  int Vpeakpeak=(n_max-n_min);
  unsigned long dvindt=Vpeakpeak/Steps;
  int n_pwm=(n_min+n_max)/2;
  unsigned long n_pexiodi=tempo/periodo;
  for(float j=0 ; j<tempo ; j=j+periodo/Steps)
  {
    //sin(rad):[-1,1]
    n_pwm=n_pwm+dvindt;
    analogWrite(OUT,n_pwm);
   delayMicroseconds(1000*periodo/Steps);
    if (n_pwm > n_max || n_pwm < n_min)
      dvindt=-dvindt;
  }
}


void setup() {
  // put your setup code here, to run once:
 pinMode(LR1,OUTPUT);
 pinMode(LR2,OUTPUT);
 pinMode(LR3,OUTPUT);
 pinMode(LR4,OUTPUT);
 pinMode(LR5,OUTPUT);

 pinMode(P1,INPUT_PULLUP);
 pinMode(P2,INPUT_PULLUP);
 pinMode(P3,INPUT_PULLUP);
 pinMode(P4,INPUT_PULLUP);
 pinMode(P5,INPUT_PULLUP);
 analogWriteResolution(12);
}

void loop() {
  // put your main code here, to run repeatedly:
  //inizializzazioni
  analogWrite(OUT, 2048);
 digitalWrite(LR1,LOW);
 digitalWrite(LR2,LOW);
 digitalWrite(LR3,LOW);
  digitalWrite(LR4,LOW) ;
```

```
  digitalWrite(LR5,LOW);
   // Verifico se l'utente ha premuto il bottone l
  if(!digitalRead(P1))
   {
     // Aspetto che l'utente rllasci ll pulsante
    while(!digitalRead(P1));
     // Aumento ll count del bottone
    if(countButton<=3)
      countButton=countButton+1;
     else
      countButton=0;
   }
   // Verlfico se l'utente he premuto il bottone 2
  if(!digitalRead(P2))
   {
     // Aspette che l'utente rilesci il pulsante
    while(!digitalRead(P2));
     // Aumente il count del bettene
   //if(countButton<=3)countButton=countButton+l;
    //else countButton=O;
    countButton=4;
   }
   // In base alle state del bcttone scelgo l'azl0ne del led
  switch(countButton)
   {
     // Faccic salixe il veltaggio
     case 1:
       //Stert Increasing Veltege
       digitalWrite(LR1 ,HIGH);
       digitalWrite(LR2 ,LOW);
       digitalWrite(LR3,LOW);
       digitalWrite(LR4,LOW);
       digitalWrite(LR5,LOW);

       for(int
IncreasingStepCounter=1;IncreasingStepCounter<=IncreasingStep;
IncreasingStepCounter++)
       {

Vapplied=IncreasingStepCounter*WorkingVoltage/mVoltForBit/Incr
easingStep;
```

```
        sinusoidale (125,300000,2048+Vapplied,2048-Vapplied);
       }
      countButton=2; //mando allo step successivo
    break;
    case 2:
      digitalWrite(LR2, HIGH);
      digitalWrite(LR1,LOW);
      digitalWrite(LR3,LOW);
      digitalWrite(LR4,LOW);
      digitalWrite(LR5,LOW);
     Vapplied=WorkingVoltage/mVoltForBit/2;
     sinusoidale(GrowthPeriod,1000,2048+Vapplied,
2048-Vapplied);

     digitalWrite(LR1, !digitalRead(LR1));
    break;
    // Stacco le GUV in lecp
    case 3:
      digitalWrite(LR3, HIGH);
      digitalWrite(LR1,LOW);
      digitalWrite(LR2 ,LOW) ;
      digitalWrite(LR4,LOW);
      digitalWrite(LR5,LOW);
     Vapplied=WorkingVoltage/mVoltForBit/2;
     quadra (250,1000,2048+Vapplied,2048-Vapplied);
    break;
    case 4:
      //Start Increasing Vcltage
      digitalWrite(LR4,HIGH);
      digitalWrite(LR1,LOW);
      digitalWrite(LR2,LOW);
      digitalWrite(LR3,LOW);
      digitalWrite(LR5,LOW);
      for(int
IncreasingStepCounter=1;IncreasingStepCounter<=IncreasingStep;
IncreasingStepCounter++)
       {
        digitalWrite(LR1,!digitalRead(LR1));
        quadra(2, 300000, 2048+ Vapplied,2048-Vapplied);
       }
     quadra(2,5400000,2048+Vapplied,2048-Vapplied);
```

```
      for(int
IncreasingStepCounter=1;IncreasingStepCounter<=IncreasingStep;
IncreasingStepCounter++)
      {
       digitalWrite(LR1,!digitalRead(LR1));
      quadra(IncreasingStepCounter+2,300000,2048+Vapplied,
2048-Vapplied);
      }
      countButton=0;
    break;


    //led si spegne e la tensione si azzera
    case 0:
      digitalWrite(LR1,LOW);
      analogWrite(OUT,2048);
      delay(1000);
    break;
  }
}
```
